# Urinary and Recombinant Follicle-Stimulating Hormone in Supporting Primate Follicular Development In Vitro

**DOI:** 10.3390/cimb48090872

**Published:** 2026-08-28

**Authors:** Fuhua Xu, Shally Ibrahim, Jing Xu

**Affiliations:** 1 NW Institute of Assisted Reproduction, Portland, OR 97229, USA; 2 Division of Reproductive & Developmental Sciences, Oregon National Primate Research Center, Oregon Health & Science University, Beaverton, OR 97006, USA; wolfsh@ohsu.edu; 3 Department of Biology & Chemistry, School of Health Sciences, Liberty University, Lynchburg, VA 24515, USA; jxu8@liberty.edu

**Keywords:** urinary FSH, recombinant FSH, preantral follicle, antral follicle, follicular development, follicle culture

## Abstract

Urinary and recombinant follicle-stimulating hormone (FSH) are used clinically for ovarian stimulation. However, data on the efficacy of these two products are inconsistent. To examine the direct effects of urinary and recombinant FSH on follicular development and function, experiments were conducted using in vitro follicle maturation approach. Preantral follicles were isolated from ovaries of rhesus macaques and randomly assigned to three groups for individual culture, including recombinant human FSH alpha (rFSHa) and beta (rFSHb) and urinary human FSH (uFSH) supplementation. Follicle survival, growth, and cell-specific gene expression, as well as media estradiol, progesterone, and anti-Müllerian hormone (AMH) concentrations, were assessed. At week 5, antral follicle survival rates were higher in the uFSH group than rFSHa and rFSHb groups. Percentages of follicles with a diameter of >1 mm were greater in the uFSH than rFSHa group. Media concentrations of estradiol, progesterone, and AMH were comparable among groups. Bone morphogenetic protein 15 and growth differentiation factor 9 mRNA levels were higher in rFSHb-treated than rFSHa-treated follicles. Data suggest that, although in vitro follicle maturation can proceed despite FSH sources, urinary and recombinant protein preparations exhibit preferable effectiveness in supporting different aspects of folliculogenesis.

## 1. Introduction

Ovarian stimulation is an essential procedure to generate multiple developmentally competent oocytes capable of fertilization in assisted reproductive technology (ART) cycles, including in vitro fertilization (IVF), intrauterine insemination, and oocyte cryopreservation. During ovarian stimulation, follicle-stimulating hormone (FSH) is used to promote the development of follicles to the large antral stage when they can respond to chorionic gonadotropin to produce mature oocytes. Currently, commercial FSH products used in clinics include highly purified urinary and recombinant human proteins [1]. Studies have investigated the efficacy and safety profiles of FSH sources in clinical settings. However, data obtained are inconsistent in terms of clinical pregnancy rates, live birth rates, and ovarian hyperstimulation syndrome incidence [1,2], which could be due to the heterogeneity of patient populations, variations of ovarian stimulation protocols, influence of systemic factors including uterine activity, and molecular nature of FSH protein.

FSH produced by the anterior pituitary consists of α- and β-subunits which are specifically glycosylated with N-glycans. In humans, while the α-subunit glycosylation occurs at both αAsn^52^ and αAsn^78^, β-subunit can have partial or full N-glycan decoration at βAsn^7^ and/or βAsn^24^. A mixture of FSH isoforms has been identified in the human pituitary extracts, including FSH18, FSH21, and FSH24 which represent N-glycosylation at βAsn^7^ only, βAsn^24^ only, and both βAsn residues, respectively [3]. In addition to this so-called “macroheterogeneity”, the highly variable N-glycan structures, such as different glycan branch numbers and terminal residues, further increase the complexity of FSH molecules which is referred as “microheterogeneity”. The oligosaccharide structures include sialylated and sulfated variants of neutral core glycans. Traditional highly purified FSH, urofollitropin, is a natural preparation derived from the urine of women. Evidence suggests that urofollitropin retains macroheterogeneity similar to that of pituitary FSH despite the extensive purification processes, although microheterogeneity may vary [4,5].

To ensure a uniform isoform profile and batch-to-batch consistency for clinical applications, recombinant proteins have been obtained from the Chinese hamster ovary (CHO) cells transfected with human FSH. The commercial recombinant human FSH products widely used in clinics include follitropin α and β. The two products have the same amino acid sequences as natural FSH but differ in the glycosylation profile. Follitropin α consists of FSH18, FSH21, and FSH24, whereas follitropin β is known to contain only FSH24 [5,6,7]. In terms of microheterogeneity, the sialylation levels of follitropin α are lower than those of natural FSH from the pituitary or urine, which are further reduced in follitropin β [5,6,8]. Sulfated glycans are present in natural FSH and follitropin β, but not follitropin α [5,6,8]. Because of the specific heterogeneous features, urinary and recombinant FSH proteins vary in isoelectric points, structural stability, and biological activity [9,10].

To evaluate the direct action of different FSH sources on follicular development and function in a well-controlled system, experiments were conducted using a nonhuman primate model and in vitro follicle maturation approach with a goal of overcoming the challenges in clinical studies [11]. Individual rhesus macaques (*Macaca mulatta*) follicles developed in a three-dimensional culture system were exposed to urinary and recombinant FSH proteins to examine their effects on promoting follicle survival, growth, steroid hormone and paracrine factor production, as well as follicular cell- and oocyte-specific gene expression, thereby precluding inter-follicle and systemic effects.

## 2. Materials and Methods

### 2.1. Ovary Collection

The general care and housing of rhesus macaques was provided by the Division of Comparative Medicine, Oregon National Primate Research Center (ONPRC), Oregon Health & Science University [12]. Ovaries were collected from adult macaques (10–14 years old, *n* = 4) by the Pathology Services Unit via the ONPRC tissue distribution program [11]. Euthanasia was due to reasons unrelated to reproductive health, such as chronic colitis and reactive arthritis. Ovaries were transferred to the laboratory at 37 °C within 30 min in an HEPES-buffered holding media (CooperSurgical, Trumbull, CT, USA) supplemented with 2% (*v*/*v*) human serum protein supplement (CooperSurgical).

### 2.2. In Vitro Follicle Maturation Supported by FSH

In vitro follicle maturation was conducted using a matrix-free three-dimensional culture system [13]. Preantral follicles (diameter = 156–264 μm) were mechanically isolated from the ovarian cortex and cultured individually in the ultra-low attachment microplates (Corning, Glendale, AZ, USA) containing 200 μL alpha minimum essential medium (ThermoFisher Scientific, Waltham, MA, USA) supplemented with 6% (*v*/*v*) human serum protein supplement, 0.5 mg/mL bovine fetuin, 5 μg/mL insulin, 5 μg/mL transferrin, 5 ng/mL sodium selenite, and 10 μg/mL gentamicin (MilliporeSigma, Burlington, MA, USA). Follicles from each of the 4 animals were randomly assigned to 3 treatment groups (16 follicles/animal/group) and cultured with 15 mIU/mL of an FSH product at 37 °C and 5% CO_2_ for 5 weeks: (a) follitropin β (rFSHb; Puregon; Merck, Rahway, NJ, USA; standard primate follicle culture protocol serving as the control group) [11], (b) follitropin α (rFSHa; Gonal-f; Merck; provided by Livzon, Zhuhai, Guangdong, China), and (c) urofollitropin (uFSH; Follitron; Livzon). Follicle survival, growth, and antrum formation were assessed weekly by microscopy [13]. Culture media (100 µL) was replaced every other day. Follicular atresia was confirmed if the oocyte degenerated or was extruded from the follicle. Follicle diameters were measured using the ImageJ software (V1.53, National Institutes of Health, Bethesda, MD, USA).

### 2.3. Follicular Hormone Production and Gene Expression

Hormone production and gene expression were analyzed for antral follicles over 1 mm in diameter at the end of culture week 5, when in vitro-developed macaque follicles could respond to human chorionic gonadotropin (hCG) stimulation to achieve oocyte maturation [13]. Culture media samples were collected and measured for concentrations of steroid hormones, estradiol and progesterone, using chemiluminescent assay and anti-Müllerian hormone (AMH) using enzyme-linked immunosorbent assay (AL-105; AnshLabs, Webster, TX, USA) by the Endocrine Technologies Core at ONPRC [11,13].

In vitro-developed antral follicles with a diameter over 1 mm were harvested at the end of culture week 5 and pooled by animal and treatment. Total RNA was extracted using the Absolutely RNA Nanoprep Kit (Agilent Technologies, Santa Clara, CA, USA) and reverse-transcribed into cDNA using the GoScript Reverse Transcription System (Promega, Madison, WI, USA) according to the manufacturers’ protocols. Real-time PCR was conducted using the TaqMan Gene Expression Assays and Applied Biosystems 7900HT Fast Real-time PCR System (ThermoFisher Scientific) to assess mRNA levels of factors that are critical for gonadotropin signaling (FSH receptor, *FSHR*, Assay ID: Rh01026045_m1; luteinizing hormone/choriogonadotropin receptor, *LHCGR*, Assay ID: Hs00896336_m1), cumulus cell glycolysis (platelet-specific phosphofructokinase, *PFKP*, Assay ID: Hs00737354_m1), and oocyte quality (bone morphogenetic protein 15, *BMP15*, Assay ID: Hs00193764_m1; growth differentiation factor 9, *GDF9*, Assay ID: Hs03986126_s1). Mitochondrial ribosomal protein S10 (*MRPS10*, Assay ID: Rh02983847_g1) served as internal control.

### 2.4. Statistical Analysis

Statistical analysis was performed using the SAS software (V9.4, SAS Institute, Cary, NC, USA). Data represent four individual animals in all experiments. Due to limited sample size and uncertainty of distribution for formal statistical hypothesis testing, a permutation test was used to compare proportions of surviving follicles and follicles with >1 mm diameters, and follicular gene expression between groups. One-way ANOVA for the permuted group memberships followed by pair-wise comparison between groups was performed to generate pseudo distributions of group differences. Antral follicle diameters and media hormone concentrations were analyzed for each individual follicle. Due to the skewed distributions, Kruskal–Wallis test followed by pair-wise mean rank test, with each follicle as an experimental unit, were performed to evaluate treatment effects. Differences were considered significant at *p* < 0.05 and values are presented as mean ± SEM.

## 3. Results

### 3.1. Macaque Follicular Development In Vitro

**Figure 1:**
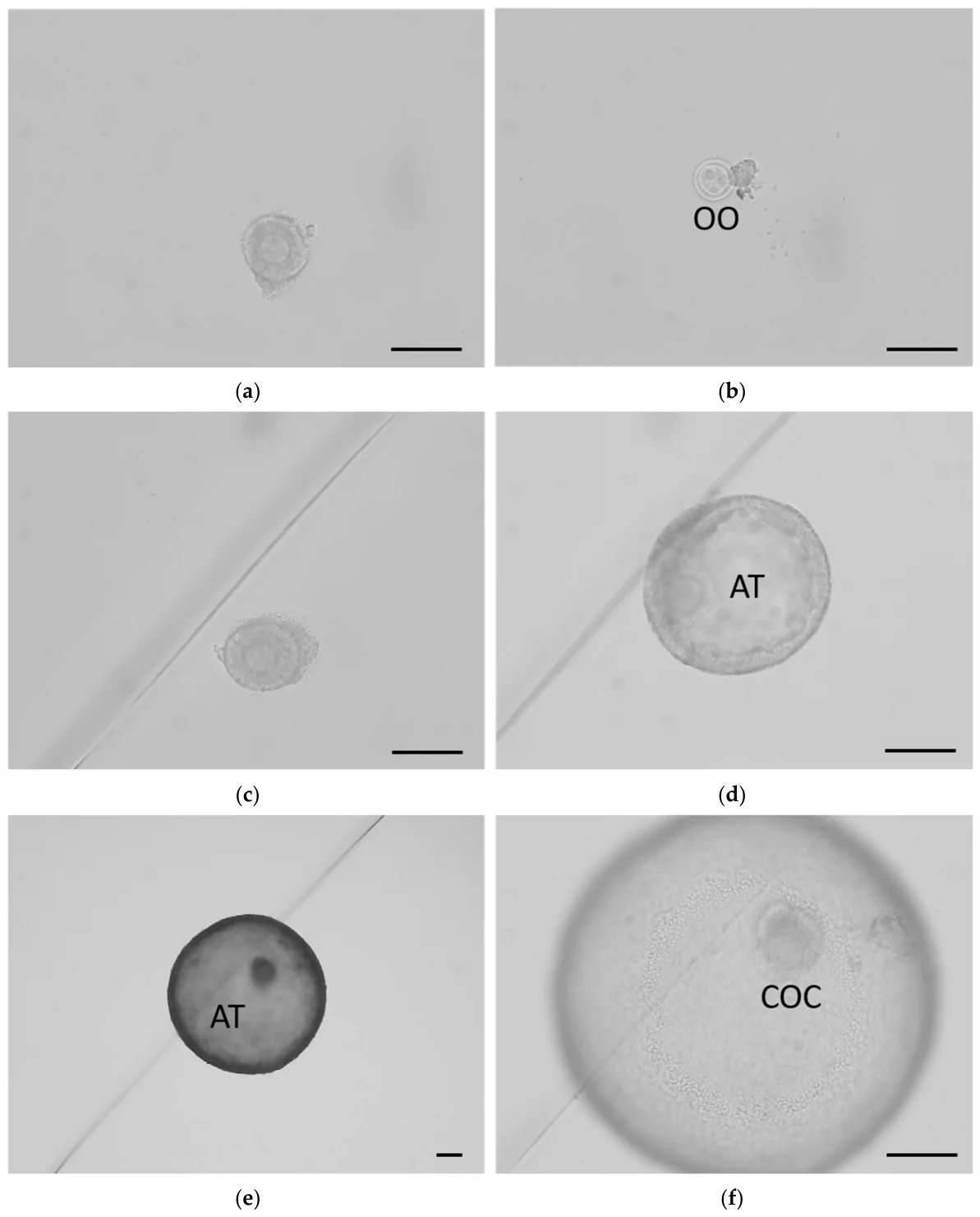
In vitro maturation of macaque follicles. With urofollitropin supplementation, some preantral follicles (**a**) underwent atresia after antrum formation at culture week 3 with degenerated oocyte extruded (**b**). Other preantral follicles (**c**) survived after antrum formation at week 3 (**d**) with diameters increased markedly at week 5 (**e**). When focusing on the cumulus–oocyte complex in the antral follicle, a healthy oocyte surrounded by cumulus cells was visible (**f**). AT, antrum; COC, cumulus–oocyte complex; OO, oocyte. Scale bar = 200 μm.

Similar patterns of in vitro development were observed in cultured macaque follicles despite FSH sources. Using the uFSH group as an example, individually cultured macaque preantral follicles exhibited a centrally located oocyte surrounded by layers of granulosa cells (Figure 1a). Antrum formation was observed in culture week 3. Some in vitro-developed antral follicles underwent atresia as indicated by vacuolated or condensed oocyte cytoplasm. The degenerated oocyte was often extruded from the follicle (Figure 1b). In contrast, although starting from the same preantral stage (Figure 1c), other follicles survived and maintained the three-dimensional architecture after antrum formation in culture week 3 (Figure 1d). The follicle diameter increased markedly at the end of culture week 5 due to enlarged antral cavity filled with follicular fluid (Figure 1e). The cumulus–oocyte complex consisting of a healthy oocyte surrounded by layers of cumulus cells was visible close to the follicle wall on the side of the antrum (Figure 1f).

### 3.2. Antral Follicle Survival and Growth

**Figure 2:**
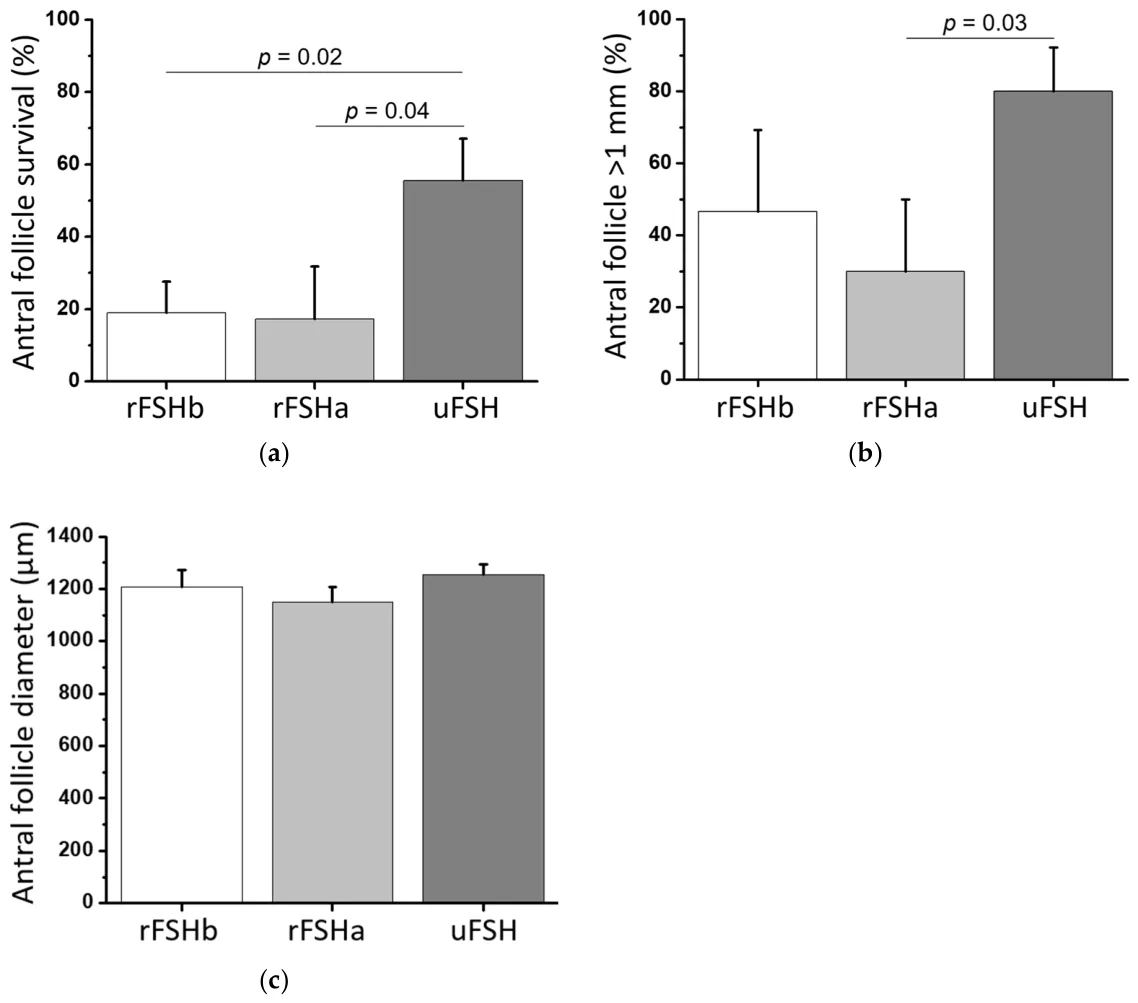
Survival and growth of macaque antral follicles developed in vitro. Antral follicle survival rates were higher in the uFSH group than those of the rFSHb or rFSHa group (*n* = 4 animals/group) (**a**). The percentages of antral follicles over 1 mm in diameter were higher in the uFSH group than those of the rFSHa, but not the rFSHb, group (*n* = 4 animals/group) (**b**). The antral follicle diameters were comparable between groups (*n* = 6, 5, and 10 follicles for rFSHb, rFSHa, and uFSH group, respectively) (**c**). Values are presented as mean ± SEM. rFSHa, follitropin α; rFSHb, follitropin β; uFSH, urofollitropin.

At the end of culture week 5, antral follicle survival rates were calculated as the percentage of surviving antral follicles over total antral follicles formed at week 3. Follicle survival rates were higher (*p* < 0.05) in the uFSH group than those of the rFSHb and rFSHa groups (Figure 2a). Despite the FSH sources, some in vitro-developed macaque antral follicles achieved a diameter over 1 mm acquiring the potential of oocyte maturation in response to hCG stimulation [13]. The percentages of hCG-responsive follicles over total surviving antral follicles were higher (*p* < 0.05) in the uFSH group than those of the rFSHa group, but not the rFSHb group (Figure 2b). The diameters of hCG-responsive antral follicles were comparable between culture groups (Figure 2c).

### 3.3. Follicular Hormone Production

**Figure 3:**
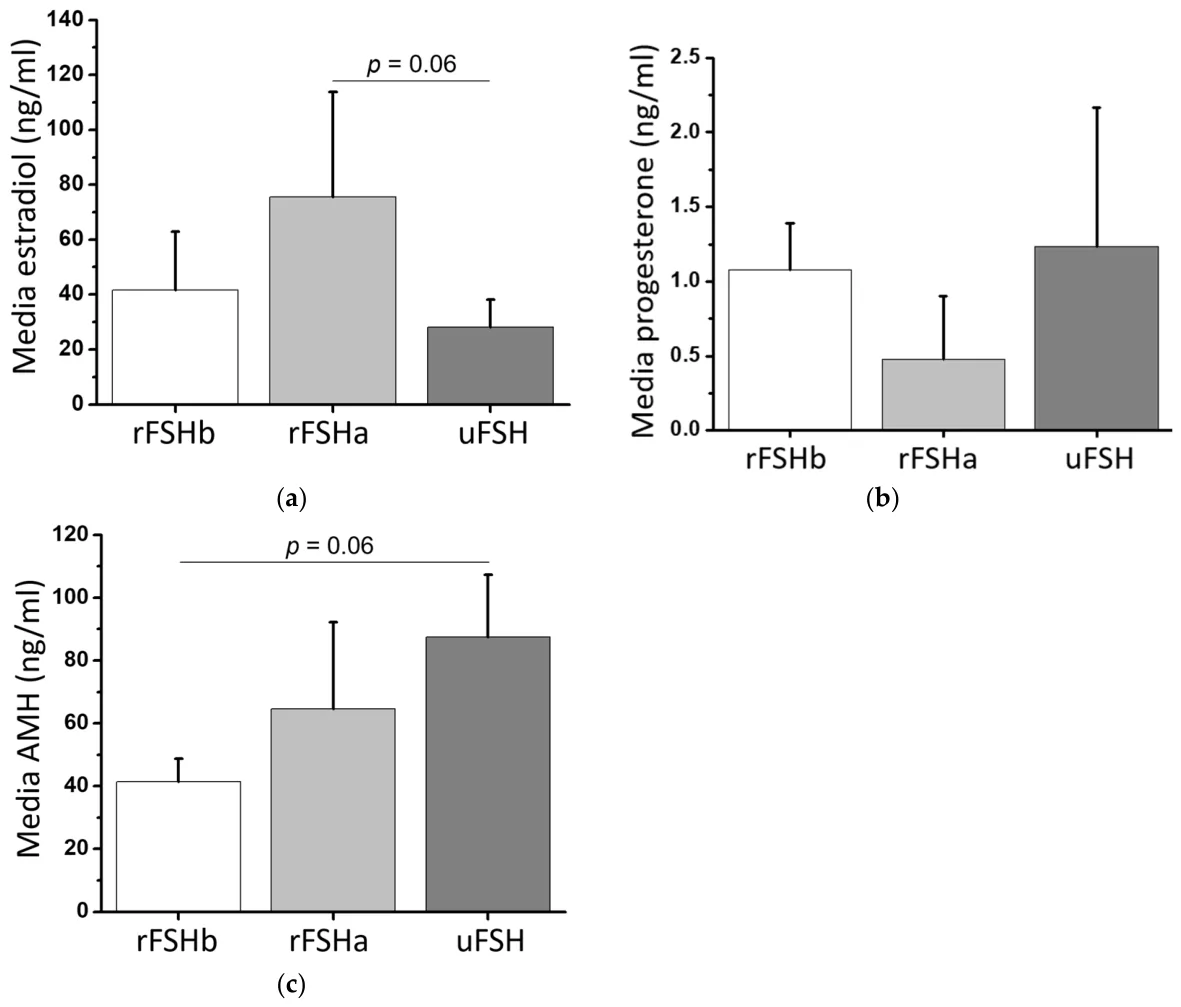
Hormone production by macaque antral follicles developed in vitro (*n* = 6, 5, and 10 follicles for rFSHb, rFSHa, and uFSH group, respectively). At the end of culture week 5, there was a trend increase in media estradiol concentrations produced by follicles treated with rFSHa compared with those treated with uFSH, but not with rFSHb (**a**). There were no differences in media progesterone concentrations between culture groups (**b**). There was a trend increase in media AMH concentrations produced by follicles cultured with uFSH compared with those cultured with rFSHb, but not with rFSHa (**c**). Values are presented as mean ± SEM. AMH, anti-Müllerian hormone; rFSHa, follitropin α; rFSHb, follitropin β; uFSH, urofollitropin.

Steroid and peptide hormones produced by in vitro-developed antral follicles were detectable in the culture media collected at the end of culture week 5. For antral follicles that were over 1 mm in diameter, although media estradiol concentrations of the rFSHb group were comparable to those of the rFSHa and uFSH groups, there was a trend increase (*p* = 0.06) in follicles cultured with rFSHa relative to those cultured with uFSH (Figure 3a). Despite FSH sources, media progesterone concentrations produced by in vitro-developed antral follicles were comparable between culture groups at week 5 (Figure 3b). There was a trend increase (*p* = 0.06) in media AMH concentrations produced by follicles cultured with uFSH compared with those cultured with rFSHb, but not with rFSHa (Figure 3c). There were no differences in media AMH concentrations between the rFSHb and rFSHa groups (Figure 3c).

### 3.4. Follicular Gene Expression

**Figure 4:**
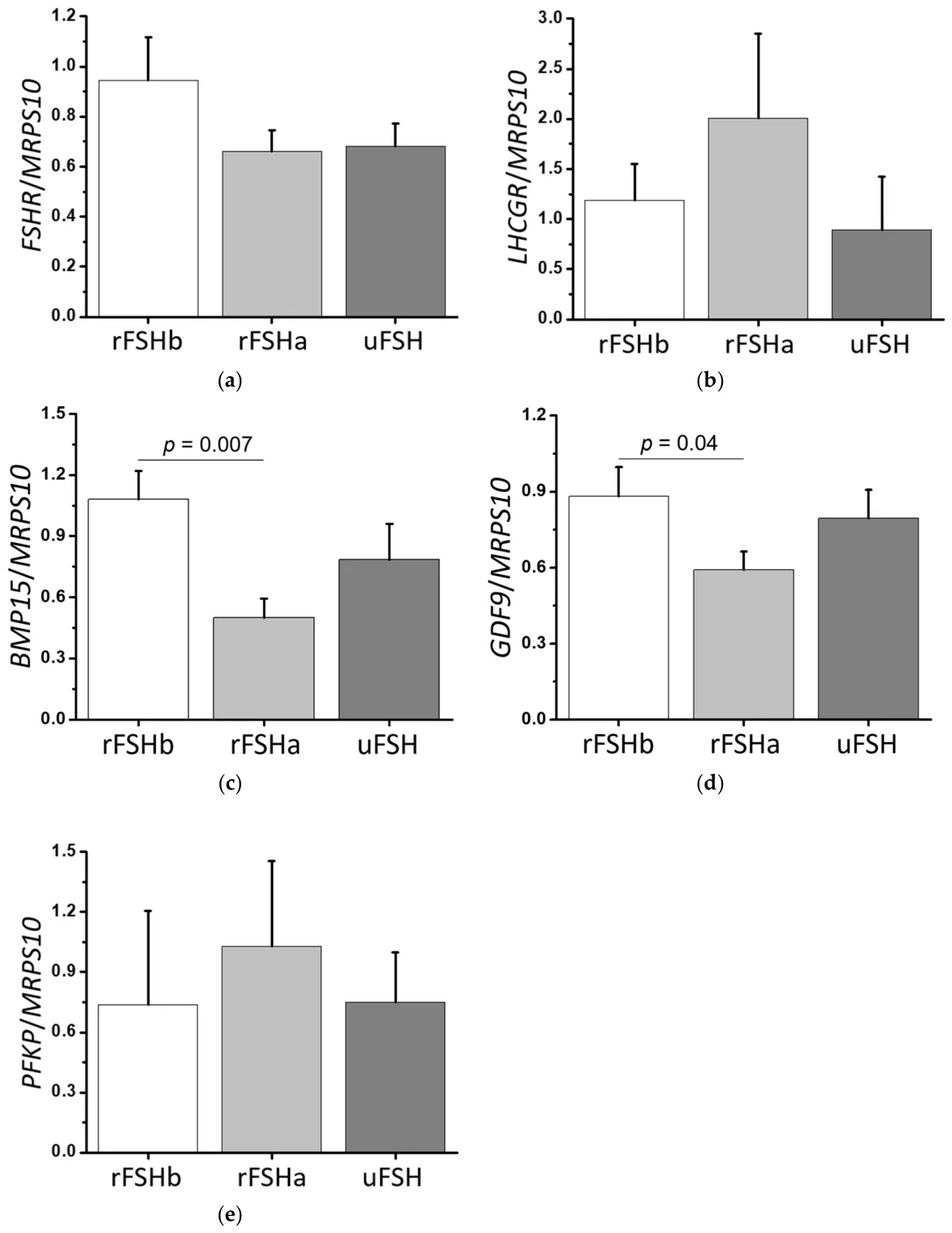
Gene expression by macaque antral follicles developed in vitro (*n* = 4 animals/group). For antral follicles that were over 1 mm in diameter, the mRNA levels of *FSHR* (**a**) and *LHCGR* (**b**) were comparable between culture groups. The mRNA levels of *BMP15* (**c**) and *GDF9* (**d**) were higher in rFSHb-treated follicles than those cultured with rFSHa, but not with uFSH. There were no differences in *PFKP* mRNA levels between culture groups (**e**). *MRPS10* served as internal control. Values are presented as mean ± SEM. *BMP15*, bone morphogenetic protein 15; *FSHR*, follicle-stimulating hormone receptor; *GDF9*, growth differentiation factor 9; *LHCGR*, luteinizing hormone/choriogonadotropin receptor; *MRPS10*, mitochondrial ribosomal protein S10; *PFKP*, platelet-specific phosphofructokinase; rFSHa, follitropin α; rFSHb, follitropin β; uFSH, urofollitropin.

The presence of *FSHR*, *LHCGR*, *BMP15*, *GDF9*, and *PFKP* mRNA was detectable in in vitro-developed antral follicles collected at the end of culture week 5. For antral follicles that were over 1 mm in diameter, the mRNA levels of *FSHR* (Figure 4a) and *LHCGR* (Figure 4b) were comparable between culture groups. However, the mRNA levels of *BMP15* (Figure 4c) and *GDF9* (Figure 4d) were higher (*p* < 0.05) in antral follicles cultured with rFSHb than those cultured with rFSHa, but not with uFSH. There were no differences in mRNA levels of *BMP15* (Figure 4c) or *GDF9* (Figure 4d) between the rFSHa and uFSH groups. Despite FSH sources, the mRNA levels of *PFKP* in antral follicles were comparable between culture groups (Figure 4e).

## 4. Discussion

Using a three-dimensional culture system, the present study investigated the direct effects of urinary and recombinant FSH products on follicular development, function, and gene expression. For the first time, the outcomes of urofollitropin, follitropin α, and follitropin β supplementation were compared in primate follicles grown individually from the preantral to antral stage. Data suggest that, although in vitro follicle maturation can proceed despite FSH sources, urinary and recombinant protein preparations exhibit preferable effectiveness in supporting different aspects of folliculogenesis.

Being a gonadotropin, FSH is essential for primate follicle culture. The same as in vivo-developed antral follicles, only some of the cultured follicles can continue to grow after antrum formation while others undergo atresia [11,12,13]. In the present study, uFSH exhibited a greater capacity in sustaining the viability of antral follicles. Compared with those exposed to rFSHa, more antral follicles responded to uFSH for further growth. Similar FSH effects on in vitro follicle growth were demonstrated in rodent studies. When mouse preantral follicles were cultured individually with urofollitropin (Fertinorm), 100% of them developed to the antral stage with diameters greater than those treated with follitropin β (Follistim) [14]. Another study cultured individual preantral follicles isolated from control mice and androgen-sterilized animals (model for anovulation) [15]. The minimal effective dose of urofollitropin (Fertinorm) was ten times lower than that of follitropin β (Puregon) in stimulating follicle growth to achieve larger diameters. Although information is limited in granulosa cells, evidence from cumulus cells has provided insight into the mechanisms of follicle survival and growth supported by different FSH preparations. In a clinical study, cumulus cells were collected from patients undergoing ovarian stimulation for IVF [16]. Compared with the follitropin (Puregon and Gonal-f) group, cumulus cells from the urofollitropin (Fostimon) group had elevated glucose transporter gene expression and carbohydrate intake, which suggested an efficient glucose metabolism supporting oocyte health and development [17]. Cumulus cells exposed to urofollitropin also exhibited enhanced lipid synthesis and metabolism which is critical for maintaining follicular metabolic homeostasis [17,18]. When cumulus cells collected from oocyte donors were analyzed, the apoptotic rates were 9% lower in the urofollitropin (Fostipur) group relative to those of the follitropin α (Gonal-f) group [19]. The observations in cumulus cells may or may not apply to granulosa cells. Nevertheless, the improved cumulus cell function and cumulus–oocyte interaction are beneficial for continued follicular development and maturation.

In vitro-developed follicles secrete steroid hormones and paracrine factors into the culture media [11]. Estradiol production continues to elevate in growing antral follicles, whereas AMH levels plateau after antrum formation and decrease as antral follicles grow larger [12]. It appeared that uFSH-treated follicles tended to produce more AMH and less estradiol relative to those exposed to rFSHb and rFSHa, respectively. Because antral follicle sizes were comparable between groups, the high AMH levels could be due to the presence of a large number of granulosa cells or the active AMH biosynthesis in the follicle. AMH could limit estradiol production since AMH is known to inhibit aromatase expression and activity [20]. As antral follicles grow larger, AMH production decreases which promotes estradiol biosynthesis [20]. While AMH secretion was not assessed, media estradiol concentrations were measured in studies using mouse in vitro follicle maturation with inconsistent results reported. Estradiol levels produced by antral follicles developed in three-dimensional culture were comparable between the urofollitropin (Fertinorm) and follitropin β (Puregon) groups [15]. However, estradiol levels produced by follitropin β (Follistim)-treated follicles were higher than those of the urofollitropin (Fertinorm) group in two-dimensional follicle culture [14]. Similar effects of follitropin β (Puregon) versus urofollitropin (Metrodin) on estradiol production were also observed in cultured human granulosa cells [21]. It is not clear whether maintaining the three-dimensional follicle architecture and cumulus–oocyte interaction alters estradiol production in response to FSH. Controversial findings were also reported in clinical studies measuring estradiol concentrations in the follicular fluid of large antral follicles developed in vivo. In patients undergoing ovarian stimulation for IVF, estradiol levels were higher in the follitropin (unknown vendor) group than those of the urofollitropin (unknown vendor) group [22]. However, estradiol levels were similar between oocyte donors receiving urofollitropin (Fostipur) and follitropin α (Gonal-f) for ovarian stimulation [19]. Nevertheless, oocyte retrieval and maturation parameters were comparable between groups in both studies [19,22]. It appears that differences between urofollitropin and follitropin in promoting steroidogenesis, if any, do not significantly impact antral follicle growth or oocyte maturation.

In the present study, FSH sources did not alter the expression levels of granulosa cell-specific genes encoding gonadotropin receptors or a cumulus cell-specific gene encoding a glycolysis enzyme. However, compared with rFSHa, rFSHb increased *BMP15* and *GDF9* expression in in vitro-developed antral follicles. These oocyte-specific growth factors are critical for sustaining oocyte-cumulus cell structural and functional relationships to support follicle growth and maturation [23]. Oocyte gene expression and maturation rates are often accessed in clinical studies. When GDF9 and BMP15 proteins were measured in the follicular fluid from oocyte donors undergoing ovarian stimulation, there were no differences between the urofollitropin (Fostipur) and follitropin α (Gonal-f) groups which is consistent with the present study [19]. Studies also found no difference in oocyte retrieval and/or maturation rates between ovarian stimulation cycles using follitropin α (Gonal-f or Ovaleap) and follitropin β (Puregon or Follistim) [24,25,26,27,28,29,30]. In contrast, data on oocytes retrieved and/or matured were inconsistent with greater [31,32,33,34], similar [35,36], and fewer [37,38] number of oocytes reported following urofollitropin (Fostimon, Fertinorm, Fertinex, or Metrodin) treatment relative to follitropin (Gonal-f or Puregon) treatment. Further studies are warranted to delineate the effects of FSH sources on stimulating follicle development to the large antral stage to achieve oocyte maturation.

To better understand the inconsistent observations from FSH sources, attentions have been drawn to the FSH heterogeneity and *FSHR* polymorphisms. Urofollitropin and follitropin α contains FSH18, FSH21, and FSH24, whereas follitropin β is made of only FSH24 [4,5,6,7]. In vitro studies suggested that hypoglycosylated FSH (FSH21 and FSH18) was more active than fully glycosylated form (FSH24) [3]. When receptor binding assays were performed using rat testis FSHR protein and CHO cells, FSH21/18 demonstrated more rapid and specific receptor binding with greater affinity relative to FSH24 [3]. Compared with FSH24, more ligand binding sites were identified in FSH21/18 [3]. Consistently, super-resolution imaging in human embryonic kidney (HEK293) cells showed faster kinetics of FSH21/18, compared with FSH24, in dissociating FSHR oligomers into monomers for activating downstream cAMP signaling [39]. In addition to bioactivity associated with macroheterogeneity, microheterogeneity of FSH regulates its bioavailability. Compared with follitropin α and β, urofollitropin contains more sialylated and sulfated glycans [5,6,8]. These glycosylation patterns decrease isoelectric point of FSH molecule which makes the protein more acidic with a prolonged half-life in vivo due to reduced metabolism and clearance rates [40]. In addition to FSH, genetic variations of *FSHR* also play a role in the receptor response to FSH. In oocyte donors with *FSHR* N680S polymorphism, the numbers of retrieved and mature oocytes were greater in individuals with SS genotype under urofollitropin (Fostipur) treatment compared with those treated with follitropin α (Gonal-f) [41]. In contrast, more oocytes were retrieved in NN-homozygous IVF patients treated with follitropin α (Gonal-f) than those receiving urofollitropin (Menopur) [42]. In vitro study using COS-1 cells transfected with *FSHR* N680S variants showed that cells with the S variant displayed higher cAMP production when stimulated with urofollitropin (Menopur) compared with follitropin α (Gonal-f) stimulation [42]. The differences between FSH sources were not observed in cells expressing the N variant [42]. Therefore, the molecular mechanism of FSH signaling requires further investigation taking *FSHR* polymorphisms into consideration.

According to findings from the present study, both urinary and recombinant FSH products can promote follicular development in a well-controlled system. The variations of follicle culture outcomes support the hypothesis that glycosylation plays an essential role in the biological activity of FSH. Due to limited sample size, current findings should be interpreted with caution. Additional in vitro studies with increased power are needed to further evaluate markers of follicular development, such as expression of genes involved in granulosa cell proliferation and differentiation, steroidogenesis, oxidative stress, and apoptosis. Functional endpoints of cultured follicles can also be assessed, including oocyte maturation and spindle morphology, fertilization potential, and developmental competence, which are clinically relevant outcomes. The effectiveness of urinary and recombinant proteins in promoting follicle growth and function in vivo warrants future investigation, especially via controlled ovarian stimulation in nonhuman primates which is similar to the ART cycle in women. Findings from these studies will provide critical insight into considering factors of FSH preparation choice, in addition to the availability and costs, for clinical protocol development to optimize ovarian stimulation in female infertility treatment.

## Data Availability

The original contributions presented in this study are included in the article. Further inquiries can be directed to the corresponding author.

## Abbreviations

The following abbreviations are used in this manuscript:

AMH	Anti-Müllerian hormone
ART	Assisted reproductive technology
AT	Antrum
BMP15	Bone morphogenetic protein 15
CHO	Chinese hamster ovary
COC	Cumulus–oocyte complex
FSH	Follicle-stimulating hormone
FSHR	Follicle-stimulating hormone receptor
GDF9	Growth differentiation factor 9
hCG	Human chorionic gonadotropin
HEK293	Human embryonic kidney cell line
IVF	In vitro fertilization
LHCGR	Luteinizing hormone/choriogonadotropin receptor
MRPS10	Mitochondrial ribosomal protein S10
ONPRC	Oregon National Primate Research Center
OO	Oocyte
PFKP	Platelet-specific phosphofructokinase
rFSHa	Recombinant human FSH alpha, follitropin α
rFSHb	Recombinant human FSH beta, follitropin β
uFSH	Urinary human FSH, urofollitropin
